# Modeling homeostatic and circadian modulation of human pain sensitivity

**DOI:** 10.3389/fnins.2023.1166203

**Published:** 2023-06-08

**Authors:** Jennifer Crodelle, Carolyn Vanty, Victoria Booth

**Affiliations:** ^1^Department of Mathematics, Middlebury College, Middlebury, VT, United States; ^2^Departments of Mathematics and Anesthesiology, University of Michigan, Ann Arbor, MI, United States

**Keywords:** thermal pain, sleep-wake flip-flop, circadian clock, sleep deprivation, jet lag, sleep homeostasis, pain sensitivity

## Abstract

**Introduction:**

Mathematical modeling has played a significant role in understanding how homeostatic sleep pressure and the circadian rhythm interact to influence sleep-wake behavior. Pain sensitivity is also affected by these processes, and recent experimental results have measured the circadian and homeostatic components of the 24 h rhythm of thermal pain sensitivity in humans. To analyze how rhythms in pain sensitivity are affected by disruptions in sleep behavior and shifts in circadian rhythms, we introduce a dynamic mathematical model for circadian and homeostatic regulation of sleep-wake states and pain intensity.

**Methods:**

The model consists of a biophysically based, sleep-wake regulation network model coupled to data-driven functions for the circadian and homeostatic modulation of pain sensitivity. This coupled sleep-wake-pain sensitivity model is validated by comparison to thermal pain intensities in adult humans measured across a 34 h sleep deprivation protocol.

**Results:**

We use the model to predict dysregulation of pain sensitivity rhythms across different scenarios of sleep deprivation and circadian rhythm shifts, including entrainment to new environmental light and activity timing as occurs with jet lag and chronic sleep restriction. Model results show that increases in pain sensitivity occur under conditions of increased homeostatic sleep drive with nonlinear modulation by the circadian rhythm, leading to unexpected decreased pain sensitivity in some scenarios.

**Discussion:**

This model provides a useful tool for pain management by predicting alterations in pain sensitivity due to varying or disrupted sleep schedules.

## 1. Introduction

Pain sensitivity varies across the 24 h day as observed in many clinical conditions (Bruguerolle and Labrecque, 2007). Experimental studies have shown that rhythmic influences on pain sensation occur regardless of whether pain responses are measured subjectively or objectively (Chapman and Jones, 1944; Davis et al., 1978; Sandrini et al., 1986; Bourdalle-Badie et al., 1990), suggesting that the daily cycle in pain responses occurs at a basic physiological level. The rhythmic modulation of pain sensitivity increases with pain intensity (Davis et al., 1978; Göbel and Cordes, 1990; Koch and Raschka, 2004) and has been detected in experiments involving a variety of different kinds of painful stimuli, including cold, heat, electrical current, pressure, and ischemia (see review in Hagenauer et al., 2017). For many pain conditions, including acute pain, sensitivity peaks during the night (Hagenauer et al., 2017). However, the phase of pain rhythmicity differs in other conditions such as for inflammatory pain which typically exhibits peak levels in the morning (Bellamy et al., 1991; Buttgereit et al., 2015).

Studies on the daily rhythm of pain sensitivity have suggested modulation by both the circadian rhythm and the homeostatic sleep drive, however few have separated their distinct influences (Lautenbacher et al., 2006; Bruguerolle and Labrecque, 2007; Finan et al., 2013). Recently, Daguet et al. (2022) conducted a highly controlled laboratory study to identify the circadian and homeostatic effects on pain sensitivity in humans. Subjects participated in a 34 h constant routine (CR) protocol, as is used for experimental measurements of circadian rhythms, that controlled light, sleep and wake activity, metabolic and other factors (Duffy and Dijk, 2002). Measurements of heat pain intensity were taken every 2 h in addition to measures of standard circadian markers including salivary melatonin, heart rate and body temperature. Over the 34 h CR, heat pain intensity exhibited an increasing cyclical pattern, particularly for higher temperature stimuli, which could be described by a linear component correlated to sleep pressure and a sinusoidal component correlated with the circadian rhythm. Their results suggest that the daily rhythm of pain sensitivity is regulated by the superposition of circadian and homeostatic processes. Questions remain, however, on how pain sensitivity is affected by disruptions to sleep homeostasis and the circadian rhythm as can occur under conditions such as recovery from sleep deprivation and jet lag.

Circadian rhythm and sleep homeostatic processes are well-known to interact to govern the timing and duration of sleep episodes. Mathematical models have been particularly useful in understanding how these processes interact, specifically under disrupting conditions. Early mathematical models, such as the classic Two Process Model, used phenomenological representations of the circadian rhythm and sleep homeostatic drive (Daan et al., 1984; Borbély et al., 2016). More recent models are based on the hypotheses of a network among wake- and sleep-promoting neuronal populations in the hypothalamus and the brain stem (Saper et al., 2005) whose activity is modulated by the hypothalamic circadian pacemaker, the suprachiasmatic nucleus (SCN) (Fuller et al., 2006). The simplest of these models describe the proposed mutually inhibitory interactions between wake- and sleep-promoting populations that govern state transitions as a sleep-wake flip-flop (Phillips and Robinson, 2007; Booth and Diniz Behn, 2014; Skeldon et al., 2014; Athanasouli et al., 2022). These models have been used to analyze effects on sleep timing and duration under multiple conditions, including for example sleep deprivation, shift work and developmentally motivated changes in sleep behavior (Phillips and Robinson, 2008; Postnova et al., 2012; Skeldon et al., 2016; Athanasouli et al., 2022).

In this paper, we introduce a dynamic mathematical model for circadian and homeostatic regulation of sleep-wake states and pain sensitivity. The model consists of a biophysically based, sleep-wake regulation network model, under modulation of a light-sensitive circadian clock model, coupled to data-driven functions for the circadian and homeostatic modulation of pain intensity. We use an exponentially increasing and decreasing function for the sleep homeostatic component of pain sensitivity to better align with the widely accepted form of the sleep homeostat (Rusterholz et al., 2010; Borbély et al., 2016). We show that the model reproduces the experimentally-observed measurements of pain sensitivity over a simulation of the 34 h CR protocol (Daguet et al., 2022). We use the model to predict dysregulation of pain sensitivity rhythms across different scenarios of sleep deprivation and circadian rhythm shifts, including entrainment to new environmental light and activity timing as occurs with jet lag and chronic sleep deprivation.

## 2. Methods

This section describes both the light-sensitive sleep-wake regulation network model used to simulate sleep and wake behavior modulated by the homeostatic sleep drive and the circadian rhythm, and the model for dynamic regulation of pain sensitivity due to interactions of components correlated with the homeostatic sleep drive and the circadian rhythm. We discuss our data-driven framework for coupling the dynamics of the pain sensitivity model to the sleep-wake regulation model and the justifications for parameter choices.

### 2.1. Sleep-wake flip-flop model

**Figure 1:**
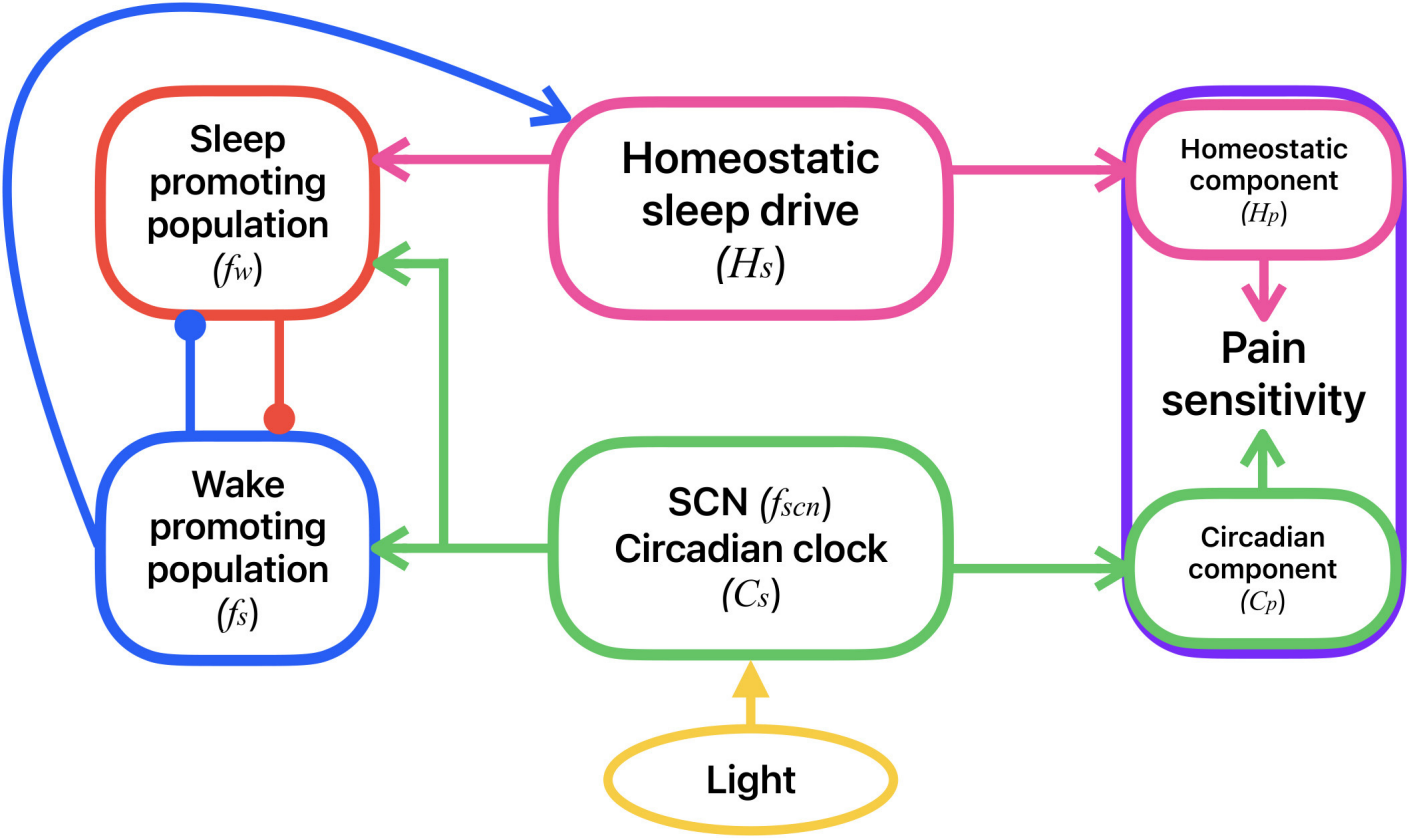
Schematic of the coupled model for the circadian and homeostatic regulation of sleep-wake behavior and pain sensitivity. Sleep-wake behavior is determined by the mutual inhibitory interactions between a sleep-promoting (red) and a wake-promoting (blue) neural population. Activity of the sleep-promoting population is influenced by the homeostatic sleep drive (magenta). Activities of both the sleep- and wake-promoting populations are modulated by the circadian clock in the SCN (green) which accepts external light input (yellow). Activities of the wake- and sleep-promoting populations dictate whether the homeostatic sleep drive is increasing (during wake) or decreasing (during sleep). Pain sensitivity (purple) consists of a homeostatic component, correlated to the homeostatic sleep drive, and a circadian component, correlated with the circadian rhythm in the SCN. Variable names for each of these model components are shown in parentheses.

Physiologically-based models of sleep-wake networks are based on the interactions of neuronal populations that promote wake and sleep states, with the suprachiasmatic nucleus (SCN) that generates the circadian rhythm (Diniz Behn et al., 2007; Phillips and Robinson, 2007; Diniz Behn and Booth, 2010; Rempe et al., 2010; Kumar et al., 2012; Gleit et al., 2013; Booth and Diniz Behn, 2014). The simplest of such ordinary differential equation (ODE) -based models consists of mutually inhibitory interactions between wake- and sleep-promoting populations, i.e., a sleep-wake flip-flop (Phillips and Robinson, 2007; Athanasouli et al., 2022). The sleep-wake flip-flop (SWFF) model used in this study includes two neuronal populations that govern the transitions between the states of wake and sleep: a wake-promoting (*W*) and a sleep-promoting (*S*) population; see Figure 1. They are coupled by mutual inhibition, and their activities are modulated by homeostatic sleep and circadian drives. In our SWFF model, the circadian input is mediated by a third neuronal population representing the suprachiasmatic nucleus (*SCN*), the neuronal population in the hypothalamus that acts as the circadian pacemaker and displays a 24-h variation in neural firing. For humans under typical conditions, the circadian rhythm and the sleep-wake cycle are entrained with lower SCN firing rates during sleep and higher SCN firing rates during wake.

We use a firing rate formalism to model the neuronal population activity. Instead of tracking the spiking of single neurons, our firing rate model describes the behavior of averaged spike rates of the neuronal populations (*f*_*W*_, *f*_*S*_, *f*_*SCN*_) (Wilson and Cowan, 1972; Deco et al., 2008). These mean firing rates in postsynaptic populations are driven by the weighted mean firing rates of their presynaptic populations.

#### 2.1.1. Neuronal populations

The equations for the neuronal populations are as follows:


(1) $$\begin{array}{l} \frac{df_{W}}{dt}=\frac{\left(W_{\infty }\left(g_{scnw}f_{SCN}-g_{sw}f_{S}\right)-f_{W}\right)}{\tau_{W}}, \end{array}$$

(2) $$\begin{array}{l} \frac{df_{S}}{dt}=\frac{\left(S_{\infty }\left(-g_{ws}f_{W}-g_{scns}f_{SCN}\right)-f_{S}\right)}{\tau_{S}}, \end{array}$$

(3) $$\begin{array}{l} \frac{df_{SCN}}{dt}=\frac{\left(SCN_{\infty }\left(C_{s}\left(t\right)\right)-f_{SCN}\right)}{\tau_{SCN}}. \end{array}$$

The postsynaptic firing rates, *f*_*X*_(*t*) (in Hz), saturate to their steady state firing rate response functions *X*_∞_(·) with time constants τ_*X*_ for *X* = *W, S, SCN*. Time *t* is in minutes. The steady state firing rate functions, *X*_∞_(·), have a sigmoidal profile, as has been utilized in many firing rate models (Wilson and Cowan, 1972; Phillips and Robinson, 2007; Deco et al., 2008; Booth and Diniz Behn, 2014):


(4) $$\begin{array}{l} \begin{array}{l} W_{\infty }(x)=W_{max}\cdot 0.5\cdot \left(1+\tanh\;\left(\frac{x-\beta_{W}}{\alpha_{W}}\right)\right), \end{array} \end{array}$$

(5) $$\begin{array}{l} \begin{array}{l} S_{\infty }(x)=S_{max}\cdot 0.5\cdot \left(1+\tanh\;\left(\frac{x-\beta_{S}(H_{s})}{\alpha_{S}}\right)\right), \end{array} \end{array}$$

(6) $$\begin{array}{l} \begin{array}{l} SCN_{\infty }(x)=SCN_{max}\cdot 0.5\cdot \left(1+\tanh\;\left(\frac{x-\beta_{SCN}}{\alpha_{SCN}}\right)\right), \end{array} \end{array}$$

where *X*_*max*_ is the maximal population firing rate, β_*X*_ is the half-activation threshold and α_*X*_ dictates the slope of the sigmoidal profile for *X* = *W, S, SCN*. The interpreted behavioral state of the model is dictated by which population, wake or sleep, has the highest firing rate.

#### 2.1.2. Homeostatic sleep drive

The homeostatic sleep drive (*H*_*s*_) regulates sleep propensity and is based on experimentally observed variation in the power of slow wave (0.75–4.5 Hz) fluctuations in electroencephalogram (EEG) recordings during sleep (Daan et al., 1984; Rusterholz et al., 2010; Borbély et al., 2016). The levels of the homeostatic sleep drive increase exponentially with the time constant τ_*hw*_ while in wake and decrease exponentially with the time constant τ_*hs*_ during sleep according to


(7) $$\begin{array}{l} \frac{dH_{s}}{dt}=\frac{H\left(f_{W}-\theta_{W}\right)\cdot \left(h_{max}-H_{s}\right)}{\tau_{hw}}+\frac{H\left(\theta_{W}-f_{W}\right)\cdot \left(h_{min}-H_{s}\right)}{\tau_{hs}}, \end{array}$$

where H represents a Heaviside function and *H*_*s*_ is in units of percent slow wave activity (SWA) power. The time constants τ_*hw*_ and τ_*hs*_ are set to experimentally estimated values for typical adult human sleep behavior (Rusterholz et al., 2010). The sleep drive *H*_*s*_ modulates the activity of the sleep-promoting population through the *H*_*s*_-dependent half-activation threshold β_*S*_(*H*_*s*_) as follows:


(8) $$\begin{array}{l} \beta_{S}\left(H_{s}\right)=k_{2}\cdot H_{s}+k_{1}. \end{array}$$

In this way as *H*_*s*_ increases during wake, the sleep promoting population eventually activates and inhibits the wake population to cause the transition to sleep. Conversely, as *H*_*s*_ decreases during sleep, the sleep population eventually inactivates and allows the wake population to activate. We define sleep onset to occur when *f*_*W*_ decreases through θ_*W*_ (and *H*_*s*_ starts to decrease) and wake onset to occur when *f*_*W*_ increases through θ_*W*_ (and *H*_*s*_ starts to increase).

#### 2.1.3. Circadian sleep drive

Twenty-four hour variation in the SCN population firing rate, *f*_*SCN*_, is driven by the human circadian clock model previously introduced in Forger et al. (1999) and Serkh and Forger (2014) and based on a modified version of the Van der Pol oscillator. Its primary variable *C*_*s*_(*t*) represents the 24-h rhythm observed in human circadian markers, specifically the core body temperature. Thus, the timing of the minimum of *C*_*s*_(*t*) represents the timing of the minimum in core body temperature. This circadian model accepts an input *I* that represents the external light level (in lux). The response of *C*_*s*_(*t*) to external light input has been fit to data on human responses to external light at different times of day (Forger et al., 1999; Jewett et al., 1999). The dynamics of *C*_*s*_(*t*) and a complementary variable, *x*_*C*_, are governed by the following equations:


(9) $$\begin{array}{ll} \frac{dC_{s}}{dt} & =\left(\frac{\pi }{720}\right)\left(x_{C}+B\right) \end{array}$$

(10) $$\begin{array}{ll} \frac{dx_{C}}{dt} & =\left(\frac{\pi }{720}\right)\left[\;\mu \;\left(x_{C}-\frac{4x_{C}^{3}}{3}\right)-C_{s}\left({\left(\frac{24}{0.99669\tau_{x}}\right)}^{2}+kB\right)\right], \end{array}$$

where μ = 0.23 represents the stiffness of the oscillator, τ_*x*_ = 24.2 h is the period of the oscillator, and *k* = 0.55 modulates the effect of the light input *B*. The model includes circadian sensitivity modulation to the external light input, described by the following equations:


(11) $$\begin{array}{l} B=\hat{B}\left(1-0.4C_{s}\right)\left(1-0.4x_{C}\right), \end{array}$$

with


(12) $$\begin{array}{l} \hat{B}=G\left(1-n\right)\alpha \left(I\right), \end{array}$$

where *G* = 33.75 and variables *n* and α govern the external light intensity *I* as follows:


(13) $$\begin{array}{ll} \alpha \left(I\right) & =\alpha_{0}{\left(\frac{I}{I_{0}}\right)}^{p}, \end{array}$$

(14) $$\begin{array}{ll} \frac{dn}{dt} & =\alpha \left(I\right)\left(1-n\right)-\beta n, \end{array}$$

where *I*_0_ = 9, 500 lux, α_0_ = 0.05min^−1^, *p* = 0.5, and β = 0.0075min^−1^.

This circadian model generates oscillations in *C*_*s*_ between −1 and 1 that can be entrained to a 24-h light:dark schedule given by *I*(*t*). The circadian drive variable *C*_*s*_(*t*) generates oscillations in the average firing rate of the SCN population, *f*_*SCN*_, between 1 and 7 Hz which is in agreement with experimental data on the neuronal activity in SCN in mammals (Meijer et al., 2010). In this paper, we consider different light schedules including the experimental light levels of the 34 h CR protocol in Daguet et al. (2022) and shifting light schedules simulating travel across time zones, as described in the Results section.

#### 2.1.4. Model parameters

The model parameter values (see Table 1) are set to generate human sleep behavior similar to experimental recordings from adults exhibiting typical sleep behavior (Benoit et al., 1980; Piltz et al., 2020). Specifically, wake and sleep durations, dictated by the time intervals when *f*_*W*_ is above or below the threshold value θ_*W*_, respectively, are 16.74 and 7.26 h for a 14:10 h light:dark cycle with lux levels 600:0. As is typical for entrained adult human sleep, wake onset occurs at the early rise of the circadian cycle, while sleep onset occurs as SCN activity approaches its minimum.

**Table 1:** Parameter values for the SWFF model.

**Wake**	***W*_*max*_ = 6 Hz**	**τ_*W*_ = 23 min**	**α_*W*_ = 0.4**	**β_*W*_ = −0.4**
Sleep	*S*_*max*_ = 6 Hz	τ_*S*_ = 10 min	α_*S*_ = 0.2	
SCN	*SCN*_*max*_ = 7 Hz	τ_*SCN*_ = 0.5 min	α_*SCN*_ = 0.7	β_*SCN*_ = −0.1
Weights	*g*_*sw*_ = 0.2508	*g*_*scnw*_ = 0.01	*g*_*ws*_ = 0.25	*g*_*scns*_ = 0.07
Homeostat	*h*_*max*_ = 323.88	*h*_*min*_ = 0	τ_*hw*_ = 946.8 min	τ_*hs*_ = 202.2 min
Circadian	*k*_1_ = −0.0118	*k*_2_ = −0.005	θ_*W*_ = 4 Hz	

For *X* = *W, S, SCN*, α_*X*_ and β_*X*_ are in units of effective synaptic input. Additionally, for *Y* = *W, S*, *g*_*XY*_ (where *X*≠*Y*) has units of (effective synaptic input / Hz). Units for *h*_*max*_ and *h*_*min*_ are percentage mean SWA. The parameters *k*_1_ and *k*_2_ are measured in effective synaptic input and effective synaptic input/(% mean SWA), respectively. The remaining units are included in the table.

### 2.2. Derivation of the homeostatic and circadian components of pain sensitivity

To model the sleep homeostatic and circadian modulation of pain sensitivity, we follow a similar approach to that described in the study by Daguet et al. (2022). In that study, the authors proposed that pain sensitivity consists of the sum of a linear homeostatic drive component and a sinusoidal circadian component. They explicitly represented these components with equations found through curve fitting to their experimental measures. Our approach also assumes that pain sensitivity can be modeled as the sum of a homeostatic drive component *H*_*p*_ and a circadian component *C*_*p*_.

To model the homeostatic component of pain sensitivity, *H*_*p*_, we replace Daguet et al.'s (2022) linear homeostatic drive with an exponential function to better correlate with the sleep homeostatic drive of our sleep-wake regulation model. We assume that *H*_*p*_ directly correlates with *H*_*s*_ such that it is modeled as an exponentially increasing function during wake and an exponentially decreasing function during sleep with the same time constants as for *H*_*s*_. The times of sleep and wake episodes are dictated by the SWFF model. The equations for the dynamic variation of *H*_*p*_ are


(15) $$\begin{array}{l} \underline{Wake:}\;H_{p}(t)=(H_{WO}-UA)\;\exp\;\left(-\frac{t-\hat{t}}{\tau_{hw}}\right)+UA,\\ \underline{Sleep:}\;H_{p}(t)=(H_{SO}-LA)\;\exp\;\left(-\frac{t-\hat{t}}{\tau_{hs}}\right)+LA, \end{array}$$

where *H*_*WO*_ and *H*_*SO*_ are the values of *H*_*p*_ at the time of wake and sleet onset, respectively, and *UA* and *LA* are the upper and lower bounds of *H*_*p*_, respectively. Since *H*_*WO*_ and *H*_*SO*_ are given as initial conditions, we have two free variables to fit to the data: *UA* and *LA*.

The study conducted by Daguet et al. (2022) collected pain sensitivity measurement data during the 34 h CR protocol during which subjects were sleep deprived. We estimated these data to provide the points used to determine the increasing *H*_*p*_ function. Using the homeostatic component of the data identified in Daguet et al. (2022) (with thermal stimulus temperature 46°C), we find a best-fit value for *UA* of 0.4125. The lower asymptote was determined as *LA* = −0.5088 by assuming that under normal sleep conditions *H*_*p*_ would return to baseline after about 8 h of sleep.

**Figure 2:**
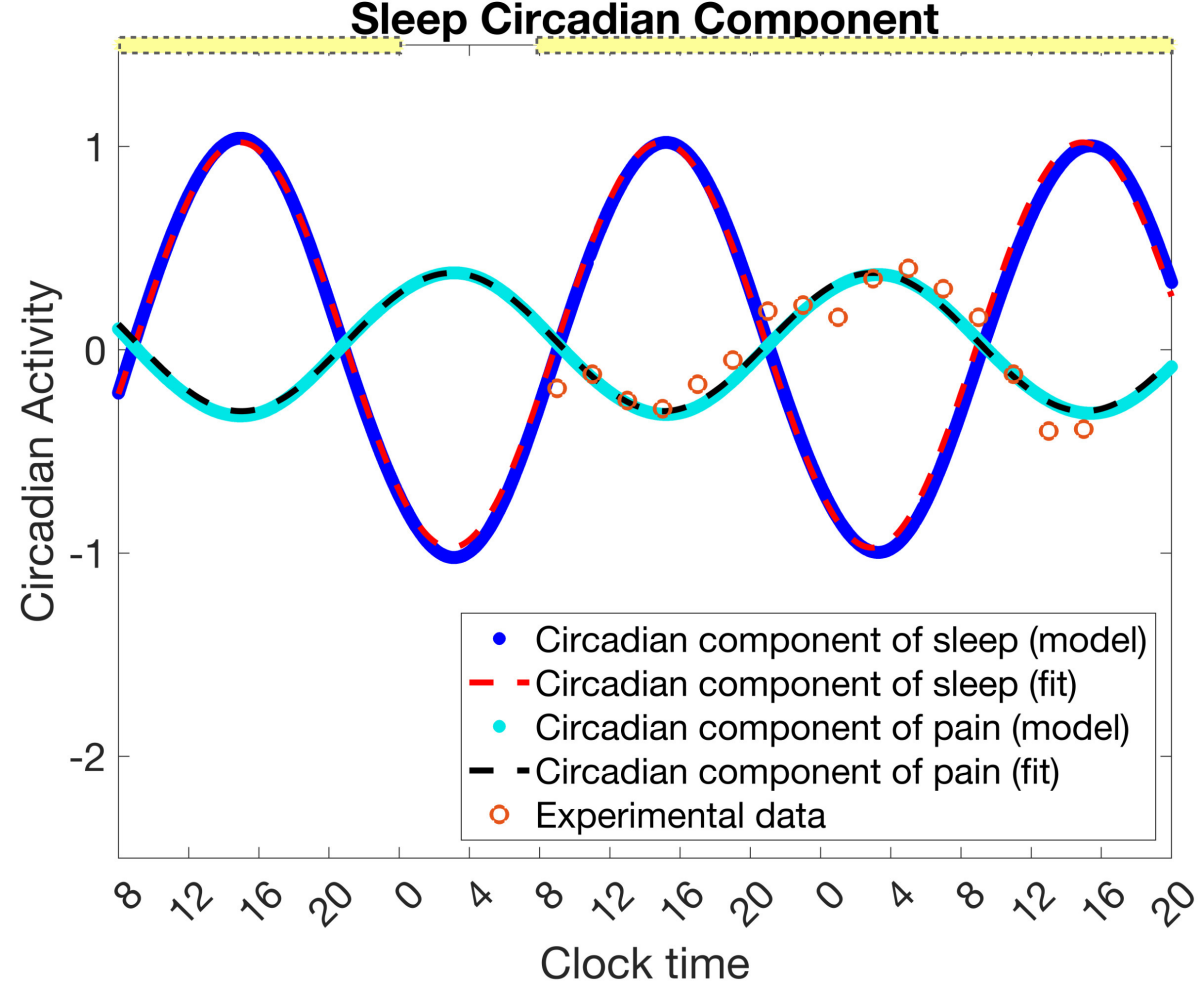
Mapping the circadian rhythm in the SCN, *C*_*s*_, to the circadian component of pain intensity *C*_*p*_. Data (orange circles) for the circadian component of pain intensity as estimated from Daguet et al. (2022) (for thermal stimuli temperature 46°C) is fit with a sinusoidal function $C_{p}^{fit}$ (Equation 16, black dashed curve). From a simulation of the experimental protocol in Daguet et al. (2022) with the SWFF model, the circadian clock of the SCN *C*_*s*_ (blue dots) is fit with a sinusoidal function (Equation 17, red dashed curve). The transformation given in Equation 18 maps $C_{s}^{fit}$ to $C_{p}^{fit}$ (cyan dots). The yellow bars indicate the timing of low light (0.5 lx), while the absence of a yellow bar indicates 0 lux. The circadian component of pain, *C*_*p*_ and its sinusoidal fit are in units of *z*-scores of pain intensity as in the data, while the circadian component of sleep, *C*_*s*_, and its sinusoidal fit are unitless.

To determine the circadian component of pain sensitivity, we fit the identified circadian component of Daguet et al.'s pain sensitivity measurement data (with thermal stimulus temperature 46°C) (Figure 2, orange circles) with a sinuosoidal function with period 24 h (1,440 min) (Figure 2, black dashed curve). The resulting equation for the fit circadian component of pain sensitivity is


(16) $$\begin{array}{l} C_{p}^{fit}\left(t\right)=0.34\sin\left(\frac{2\pi }{1440}\left(t-780\right)\right)+0.038. \end{array}$$

As described below, we assume that the circadian component of pain sensitivity is directly correlated with the circadian rhythm in the SCN, *C*_*s*_. To complete the model description, we identify a transformation from *C*_*s*_ to the circadian component of pain sensitivity, *C*_*p*_, using the fit function $C_{p}^{fit}$.

For *H*_*p*_, $C_{p}^{fit}$ and *C*_*p*_, we use the same units as in Daguet et al. (2022), namely *z*-scores of pain intensity measured by a visual analog scale.

### 2.3. Full model for pain sensitivity coupled to the SWFF model

To combine the SWFF model and pain sensitivity model, we use the wake and sleep times determined by the SWFF model to dictate the timing of switches between the increasing and decreasing, respectively, behavior of *H*_*p*_ (Equation 15). We also assume that the circadian clock of the SCN, *C*_*s*_, directly drives the circadian component of pain, *C*_*p*_. Following Daguet et al. (2022), the total pain sensitivity is the sum of *H*_*p*_ and *C*_*p*_. What remains to be described is how we map *C*_*s*_ to *C*_*p*_.

To find a mapping from *C*_*s*_ to *C*_*p*_, we simulate the SWFF model for the experimental protocol of Daguet et al. (2022), namely a first day of 16 h of wake and 8 h of sleep followed by 34 h of sleep deprivation starting on the 2nd day. The light pattern followed Daguet et al. (2022) with a laboratory light setting of 0.5 lux from 8 to 12 a.m. and zero lux from 12 to 8 a.m. on the first day, and 0.5 lux for the remaining 34 h of sleep deprivation. We then fit *C*_*s*_ simulated under these conditions (Figure 2, blue dots), with the following sinusoidal function (Figure 2, red dashed curve)


(17) $$\begin{array}{l} C_{s}^{fit}\left(t\right)=0.9968\sin\left(\frac{2\pi }{1440}\left(t-56\right)\right)+0.0235. \end{array}$$

Next, we compute a transformation from $C_{s}^{fit}$ to $C_{p}^{fit}$ by defining constant shifts in the amplitude, mid-line, and phase that map $C_{s}^{fit}$ onto $C_{p}^{fit}$ for these specific experimental conditions (Figure 2, cyan dots). Note that we set the phase shift to 12 h following the experimental finding that both the maximum of pain sensitivity (for thermal stimuli temperature of 46°C) and the minimum core body temperature both occurred at ~3:00 a.m. (Daguet et al., 2022).

We use this transformation to define how the circadian component of pain sensitivity in our model, *C*_*p*_, is correlated with the circadian rhythm driving the SCN, *C*_*s*_:


(18) $$\begin{array}{l} C_{p}\left(t\right)=0.3411C_{s}\left(t-720\right)+0.03. \end{array}$$

Finally, pain intensity, *P*(*t*) is found as the linear sum of the homeostatic pain component and the circadian pain component as follows:


$$P\left(t\right)=C_{p}\left(t\right)+H_{p}\left(t\right).$$

## 3. Results

### 3.1. Model validation

**Figure 3:**
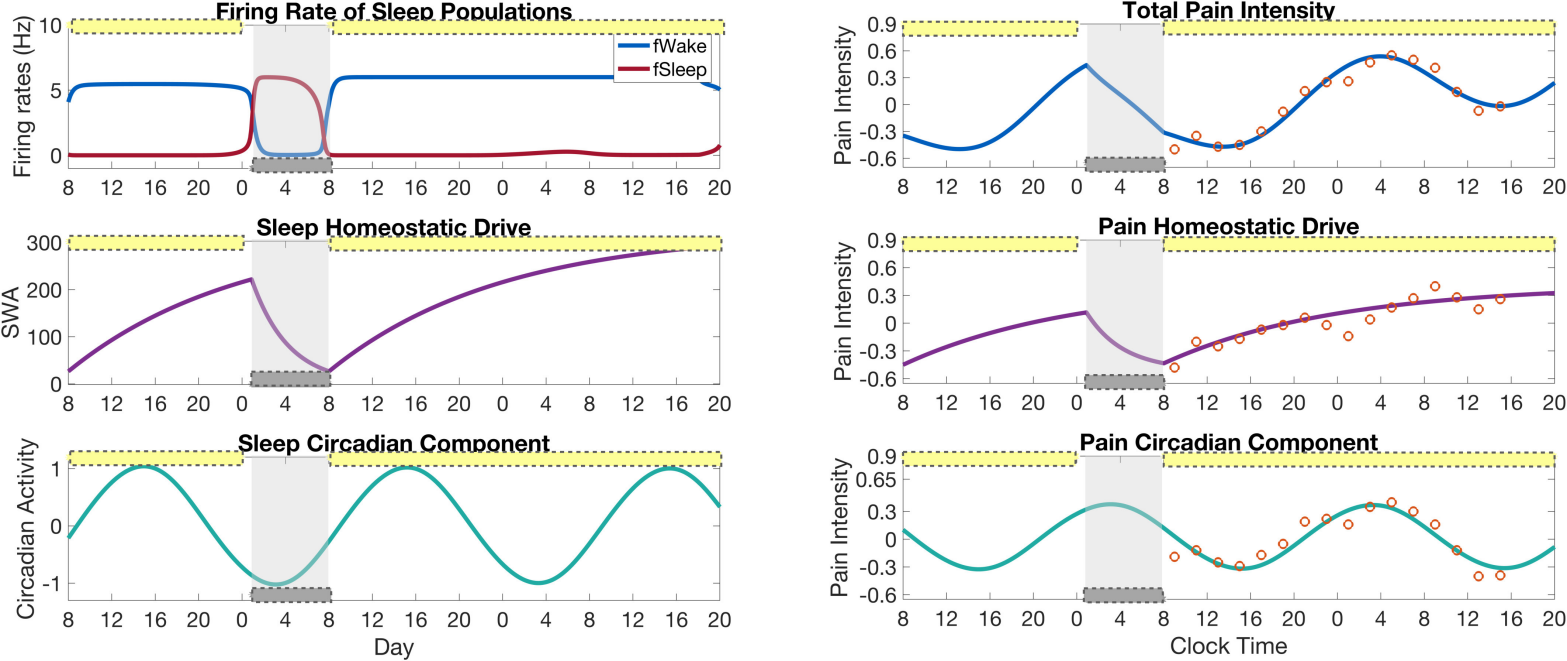
Results of the full model for pain intensity coupled to the SWFF model for a simulation of the sleep deprivation protocol in Daguet et al. (2022). **(Left panels)** Output of the SWFF model for 24 h of a “normal" day followed by 34 h of sleep deprivation. The top panel shows the firing rates of the wake (blue) and sleep (red) populations, the middle panel shows the sleep homeostatic drive *H*_*s*_ as measured in slow wave activity (SWA), and the bottom panel shows the circadian rhythm in the SCN *C*_*s*_. **(Right panels)** Output of the pain intensity model with comparison to experimental data of Daguet et al. (2022) (orange circles). The top panel shows total pain intensity computed as the sum of the homeostatic component *H*_*p*_ (middle panel) and the circadian component *C*_*p*_ (bottom panel). The yellow bars indicate the timing of low light (0.5 lx), while the gray bars indicate the timing of sleep as dictated by the SWFF model.

We first validate our model by reproducing the variation of pain sensitivity as measured by Daguet et al. (2022) over the 34 h sleep deprivation protocol. Figure 3 shows results from both the SWFF model (left panels) and the pain sensitivity model (right panels) over the 2.5 days of the CR experimental protocol. Activity of the wake- and sleep-promoting populations in the SWFF model (top left panel) replicate behavior over the first day of 16 h in wake and 8 h in sleep, followed by 34 h of sleep deprivation (forced wakefulness) in 0.5 lux. The homeostatic sleep drive *H*_*s*_ (middle left panel) rises to increased levels during sleep deprivation and the circadian rhythm in the SCN, *C*_*s*_ (left bottom panel) shows only slight modulation by the low light levels. These dynamics of *H*_*s*_ and *C*_*s*_ dictate the dynamics of the homeostatic *H*_*p*_ (right middle panel) and circadian *C*_*p*_ (right bottom panel) components of pain sensitivity, computed by Equations (15) and (18), respectively. Both *H*_*p*_ and *C*_*p*_ show good agreement with the experimental data (orange circles), as does total pain sensitivity (right top panel), computed as the sum of *H*_*p*_ and *C*_*p*_.

### 3.2. Recovery from sleep deprivation

**Figure 4:**
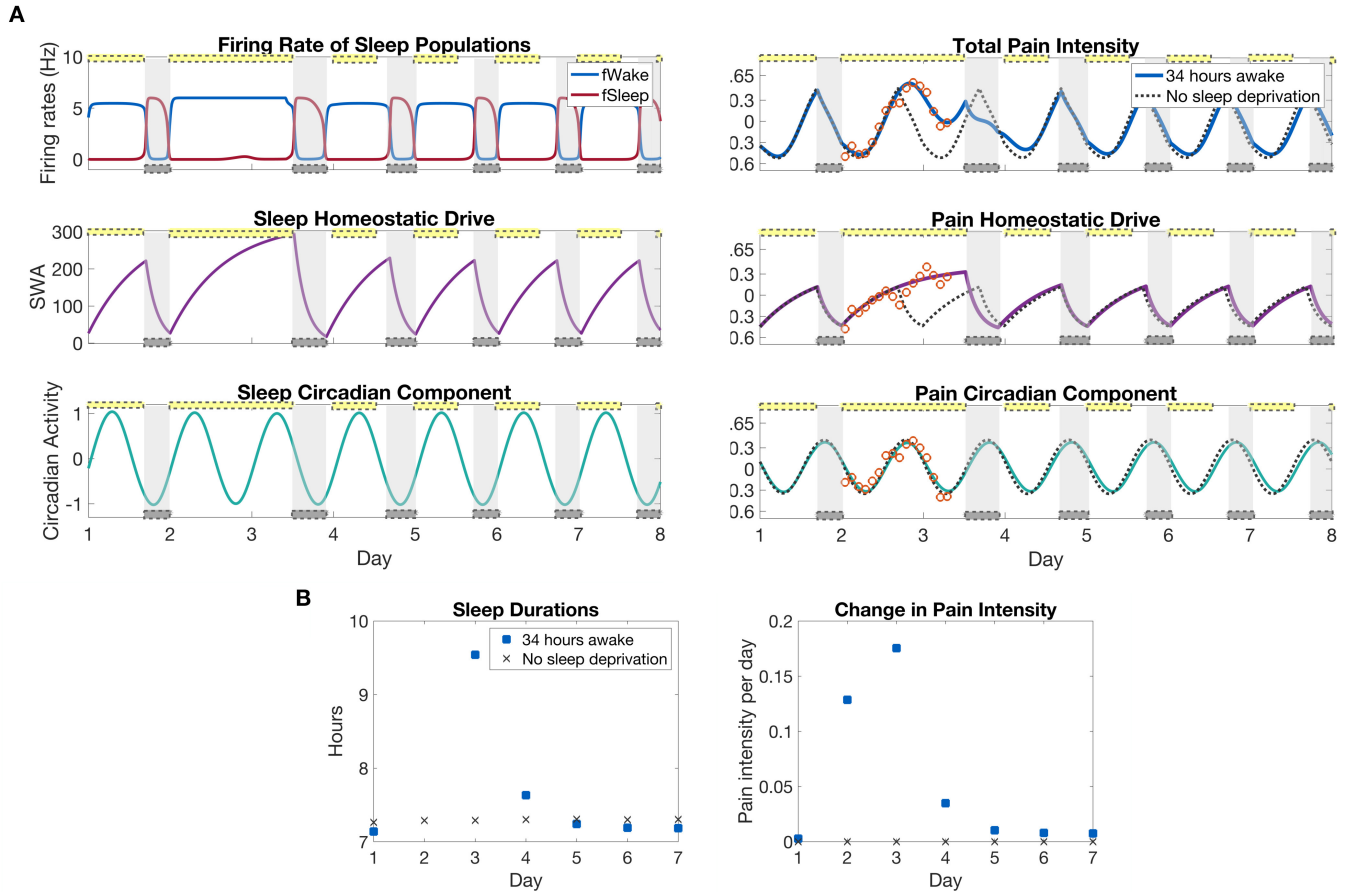
Model predictions of differences in pain sensitivity during recovery from the sleep deprivation protocol in Figure 3. **(A)**, left panels: Output of the SWFF model for the 34 h sleep deprivation protocol as in Figure 3 and the following 5 days. The top panel shows the firing rates of the wake (blue) and sleep (red) populations, the middle panel the sleep homeostatic drive *H*_*s*_, and the bottom panel the circadian rhythm in the SCN *C*_*s*_. **(A)**, right panels: Output of the pain sensitivity model for the sleep deprivation protocol (solid curves) together with a simulation with no sleep deprivation (dashed curves). The top panel shows total pain intensity, the middle panel the homeostatic component, and the bottom panel the circadian component of pain sensitivity. **(B)**, left panels: The duration of sleep that occurred each day in the sleep deprivation protocol and for the case with no sleep deprivation. **(B)**, right panels: Average change in pain sensitivity over the sleep deprivation protocol as compared to a normal simulation with no sleep deprivation. The measure is computed by taking the average over each 24 h period of the difference between total pain intensity with sleep deprivation and without (difference between solid and dashed curves in top panel). The yellow bars indicate the timing of low light (0.5 lx), while the gray bars indicate the timing of sleep as dictated by the SWFF model.

We are interested in the predictions made by the model for how pain sensitivity varies during the recovery time after the sleep deprivation protocol. For this, we continue the simulation in Figure 3 for an additional 4.5 days with a 12:12 light:dark schedule at the laboratory light setting of 0.5 lux and compute the pain intensity over the recovery days; see Figure 4A. To compute changes in pain sensitivity during the sleep deprivation protocol, we compute the average over each 24 h day of the difference between total pain intensity levels during the protocol and under normal conditions; see Figure 4B. The model predicts that pain sensitivity will be elevated during the day of sleep deprivation (day 2), with the highest pain sensitivity occurring on the day after the sleep deprivation (day 3). Pain sensitivity returns to its baseline levels by the third day after sleep deprivation (day 5). Similarly, the amount of recovery sleep is highest for the day after the sleep deprivation (day 3), with the sleep duration increasing from around 7 h in the normal sleep schedule case to almost 10 h in the sleep deprived case. The duration of each sleep bout returns to baseline levels by the third day after sleep deprivation (day 5); see Figure 4B.

**Figure 5:**
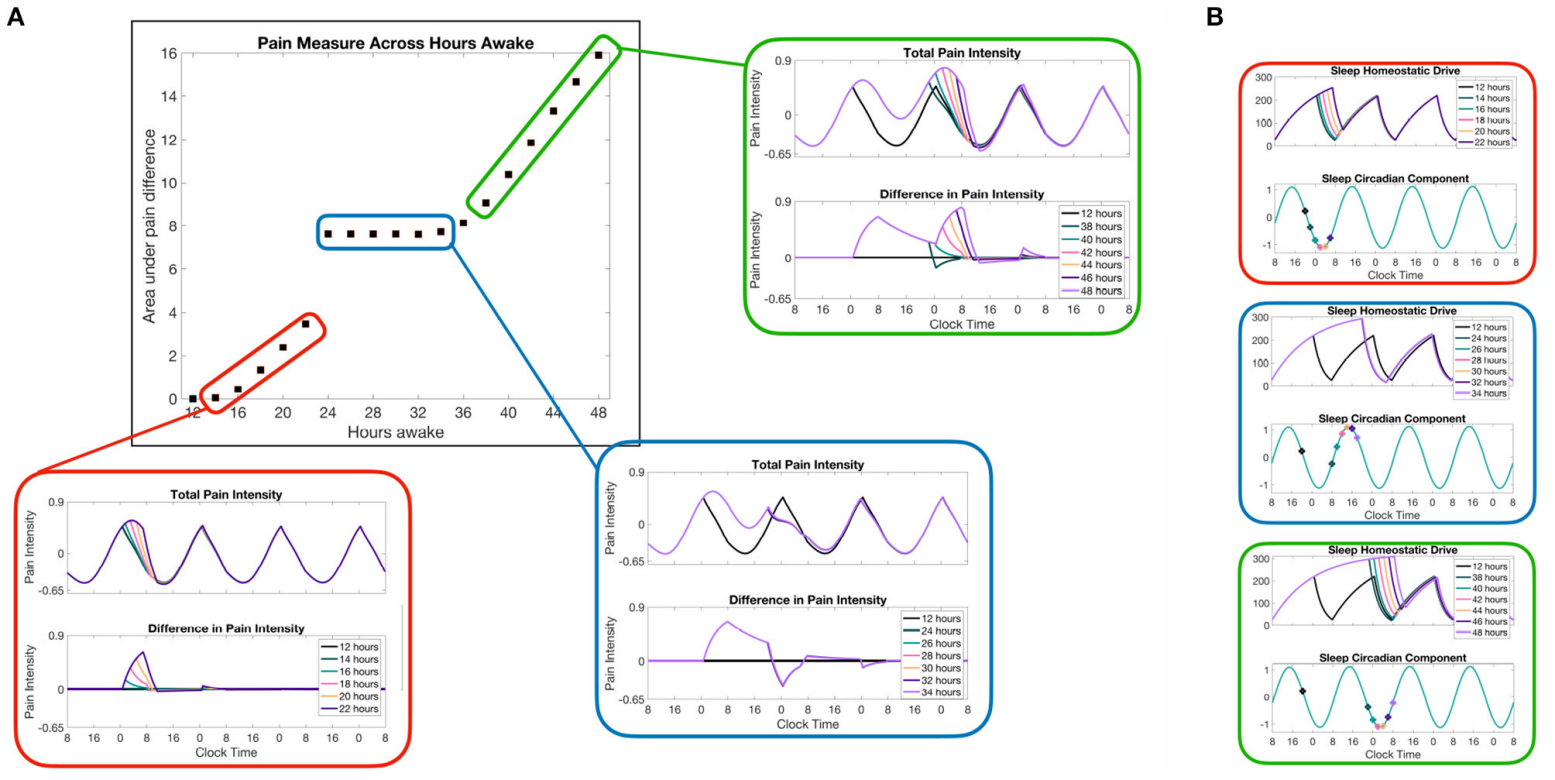
Model predictions of pain sensitivity due to different lengths of sleep deprivation. **(A)** The color-coordinated offset figures show the total pain intensity curves in the top panels and the difference in pain intensity for a normal sleep/wake schedule (no sleep deprivation) and the pain intensity resulting from each sleep deprivation case. The main panel (outlined in black) shows a measure of the total difference in pain intensity computed as the area under the difference curve across the whole simulation of 7 days and measured in units of pain intensity times hours. **(B)** Homeostatic *H*_*s*_ and circadian *C*_*s*_ variables of the SWFF model for each of the three sets of sleep deprivation duration cases. The points along *C*_*s*_ correspond to the times at which the corresponding sleep deprivation protocol (forced wakefulness) was lifted and the SWFF model was allowed to enter the sleep state.

An advantage of coupling the sleep-wake dynamics to a pain sensitivity model is the ability to predict changes in pain due to changes in the sleep schedule. As a model prediction, we used our model to measure the changes in pain sensitivity due to different amounts of sleep deprivation. In this simulation, we use a 12:12 light:dark schedule at 600 lux during light durations, simulating a more usual amount of indoor light. Following from the Daguet et al. (2022) study, we will refer to different sleep deprivation protocols by the total amount of time spent awake, including the 12 h of normal wake time. Thus, any sleep deprivation protocols from 0 to 12 h do not change the sleep or wake times at all, since the subject will have already been awake. Figure 5 shows the model results for different durations of sleep deprivation protocols. To quantify the total differences in pain sensitivity over the entire sleep deprivation protocols and the following recovery days, we subtract the total pain intensity curve for the sleep deprivation protocol case from total pain intensity over a normal daily schedule (no sleep deprivation) and take the area under the curve (middle panel). Examples of total pain intensity curves and the difference from a normal daily schedule are shown in surrounding plots (red, blue, and green).

An interesting prediction shown in the SWFF model is that any sleep deprivation duration that ends when the circadian drive to the SCN firing rate is already high (blue-highlighted results in Figure 5) doesn't result in additional pain sensitivity due to the predicted inability to fall asleep in the middle of the day, resulting in similar sleep timing. Meanwhile, for sleep deprivation durations that end during other phases of the circadian rhythm, we see significant increases in pain intensity as we increase the amount of sleep deprivation (red and green highlighted results). Another interesting prediction that occurs for sleep deprivations lasting more than 36 h is that, depending on when the sleep deprivation ends, there are times right after the sleep deprivation ends when the pain sensitivity is lower than it would be normally due to immediate sleep onset (hours earlier than usual). Overall, of course, the model predicts more pain sensitivity overall for more severe sleep deprivation cases, but for24–38 h of sleep deprivation, the model predicts lower than normal pain sensitivity for8 h following the release of sleep deprivation (blue and green highlighted results).

### 3.3. Jet lag

**Figure 6:**
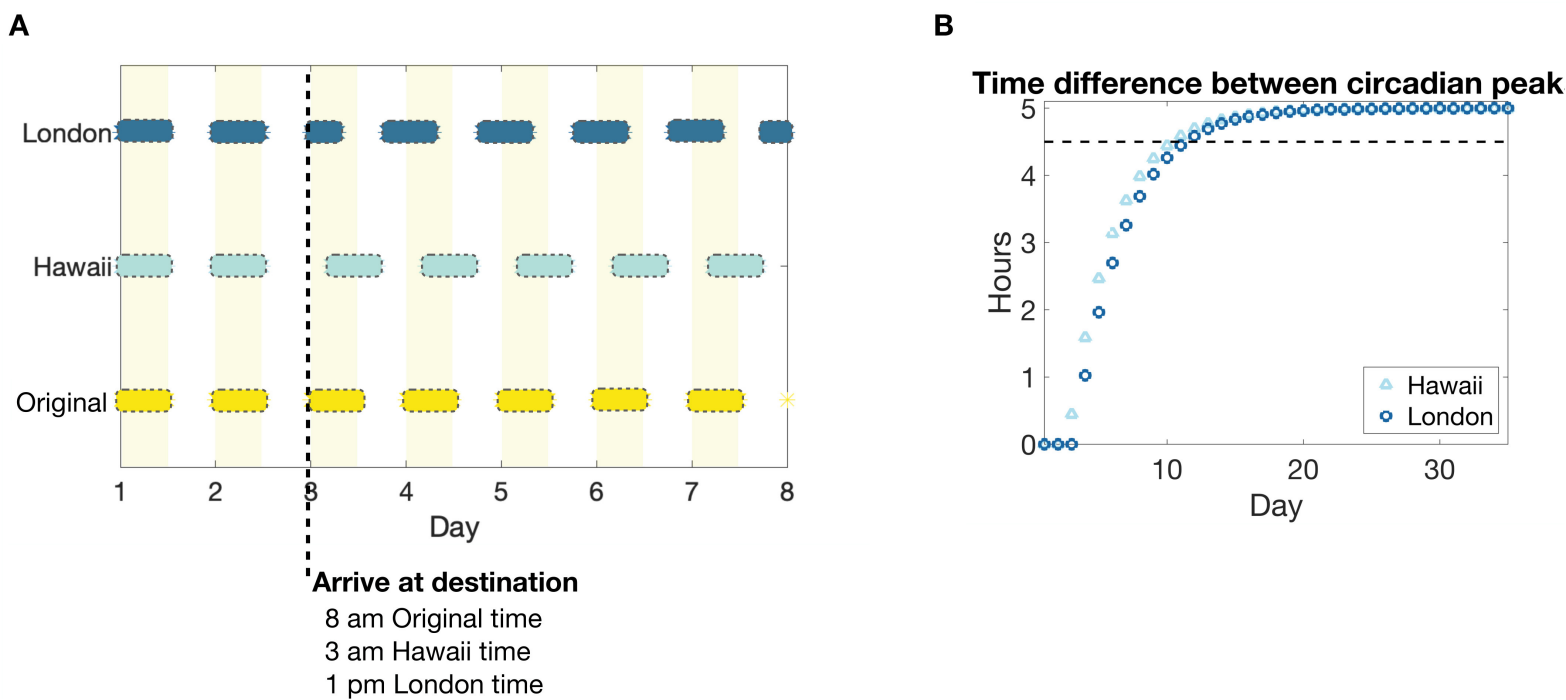
Light schedule and circadian entrainment due to eastward and westward travel of five time zones. **(A)** Light input schedule for a 5 h advance or a 5 h delay in environmental light cycle as would occur for travel to a time zone 5 h east (New York to London, dark blue bars) or to a time zone 5 h west (New York to Hawaii, light blue bars), respectively, compared to remaining in the original time zone (yellow bars and shaded regions). The colored bars correspond to periods of light in each time zone, while no bar corresponds to darkness. **(B)** The absolute value of the time difference between the peaks of the circadian drive to the SCN *C*_*s*_ in response to the east (London, dark blue circles) and west (Hawaii, light blue triangles) shifted light schedule, compared to remaining in the original light schedule. The black dotted line corresponds to a threshold of 0.5 h until the circadian rhythm is entrained to a 5 h shift from the original.

We next explore how pain sensitivity changes when there is an abrupt change in environmental light cycle, as may happen during travel to a new time zone (jet lag). In response, the circadian clock drive to the SCN will phase shift, disrupting sleep and wake times, and eventually entrain to the new light cycle. We consider two sample scenarios that result in a 5-h shift forward in time (traveling five time zones east, from New York to London, for example) and a 5-h shift backward in time (traveling five time zones west, from New York to Hawaii, for example). The simulations are set up such that a presumed traveler arrives at their destination, London or Hawaii, at 8 a.m. New York time, or 1 p.m. London time and 3 a.m. Hawaii time; see Figure 6A for the resulting light schedule.

As has been predicted in other modeling studies of circadian phase shifting (Diekman and Bose, 2018), our model predicts that circadian entrainment, as measured by the time difference between the peaks of the circadian rhythm drive to the SCN *C*_*s*_, occurs more rapidly for westward travel compared to eastward travel; see Figure 6B.

**Figure 7:**
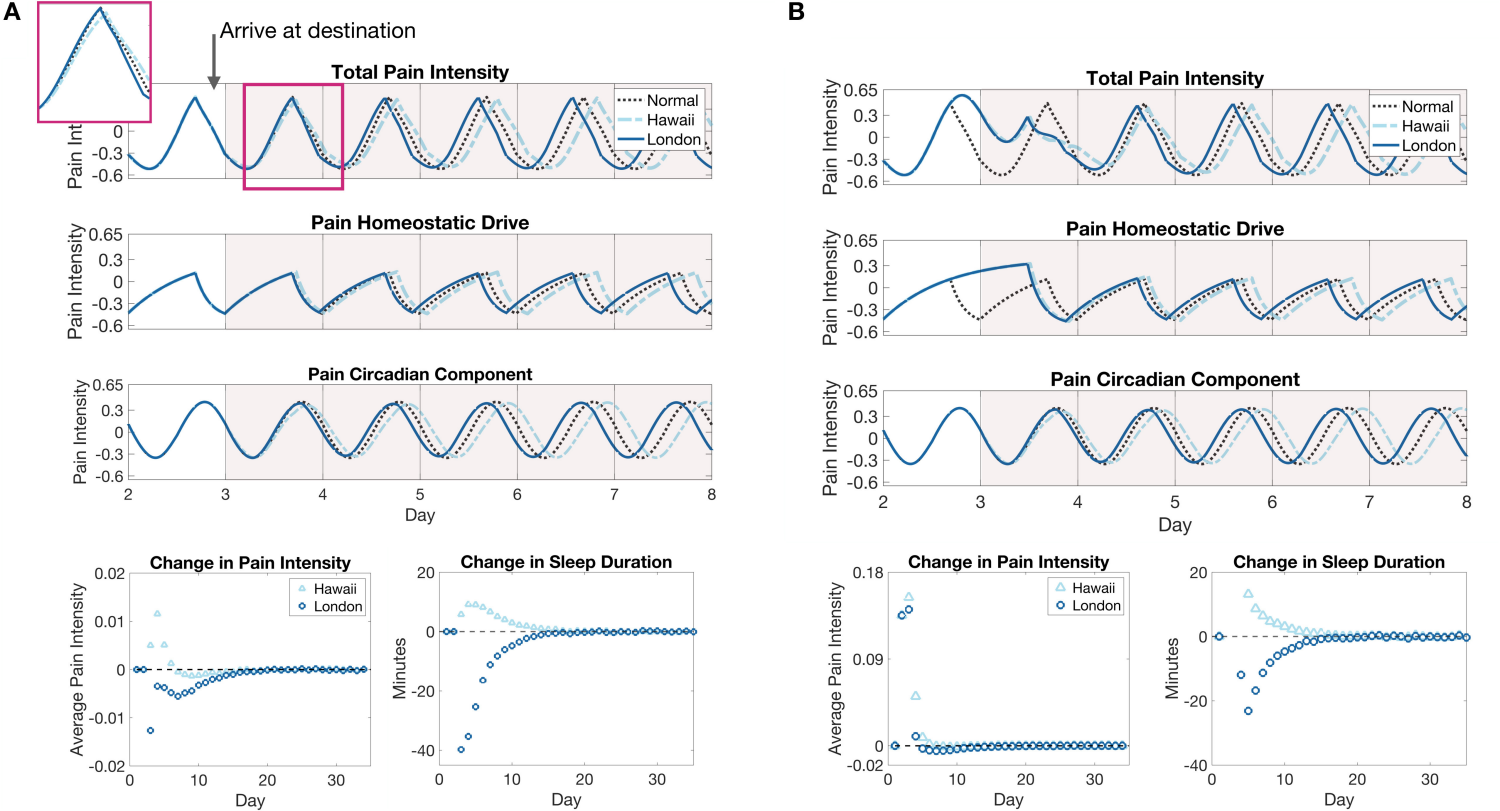
Pain sensitivity due to eastward and westward travel of five time zones without **(A)** and with **(B)** forced wakefulness during the flight to the destination. **(A)**, The top panel show the model prediction of changes in pain intensity due to a 5 h advance or a 5 h delay in environmental light cycle as would occur for travel to a time zone 5 h east (New York to London, solid blue curves) or a time zone 5 h west (New York to Hawaii, light blue dashed curves), respectively, compared to remaining in the original time zone (black dotted curve). The inset shows the pain intensity for the first day in the new time zone. The bottom panels show the daily average difference in total pain intensity between time zone shift cases and remaining in the original time zone (left panel) and the change in sleep durations on each day of the east (London, dark blue circles) or west (Hawaii, light blue triangles) shifted light cycle compared to remaining in the original light cycle (right panel). **(B)** The same plots for the jet lag simulations with forced wakefulness during the flight. The light cycle is shifted at 8 a.m. on the third day.

The shift in circadian rhythm in the new locations has interesting effects on pain sensitivity; see Figure 7A. During the shifting of the circadian rhythm to the new time zone, the model predicts a small (about 10 min) increase in the duration of sleep bouts for westward travel compared to a decrease (at most 40 min) in the duration of sleep bouts for eastward travel. Yet, despite the decrease in sleep duration for the first few days in London, the model predicts a decrease in daily average pain sensitivity. This can be explained by the backward shift in the peak of the circadian component *C*_*p*_ during the entrainment process such that it better aligns with the peak of the homeostatic component *H*_*p*_. Conversely, for travel to Hawaii, the circadian component is pushed forward during the entrainment process such that its peak coincides with lower portions of the decreasing phase of the homeostatic component, resulting in a larger than normal pain sensitivity when the two are added together. Thus, while entrainment to a westward shift (delay) in light cycle occurs more quickly with less disruption of sleep duration, it may be accompanied by increases in overall pain sensitivity, compared to the same magnitude shift in the eastward (advance) direction. Once the circadian rhythm has entrained to the new time zone, pain sensitivity returns to baseline values since sleep duration does not change in the new time zone.

### 3.4. Jet lag with sleep deprivation

In the previous section, we simulated jet lag scenarios assuming that the traveler arrived at their location with no disruption to their normal sleep-wake behavior prior to arrival. In reality, the travelers will be on a plane for some hours before arriving in their destination, oftentimes not being able to sleep or experiencing many sleep disruptions. In this section, we again simulate 5 h light cycle shifts corresponding to travel from New York to London or Hawaii, but we also simulate the required 8 or 11 h, respectively, plane ride during which the traveler is kept awake. Once the traveler reaches their destination, their sleep and wake times are again dictated by the SWFF model.

Figure 7B shows model predictions of pain sensitivity in this case. First, note that the circadian entrainment remains the same as in Figure 6B, since sleep deprivation only affects the homeostatic sleep drive *H*_*s*_. During the shift in the circadian rhythm, the amount of sleep each day is similar for both travel scenarios in the days after arrival at the respective destinations, again due to increased *H*_*s*_ (compare sleep durations in Figures 7A, B), with a slight decrease in the change in sleep durations for eastward travel in the sleep deprivation case (from about 40 min shorter to 20 min shorter as compared to the original time zone). In contrast to the previous case in which pain sensitivity decreased for eastward travel, when coupled with sleep deprivation on the flight, pain sensitivity increases drastically in both cases on the day of travel due to the high homeostatic sleep drive. During the entrainment process, pain sensitivity still remains slightly lower for eastward travel than westward travel, with a small decrease in sensitivity occurring before it returns to pre-travel levels. These results show that accounting for the combined effects of sleep deprivation and circadian rhythm shifts are important for accurate predictions of travel-induced disruptions in sleep and pain sensitivity.

### 3.5. Chronic sleep deprivation

**Figure 8:**
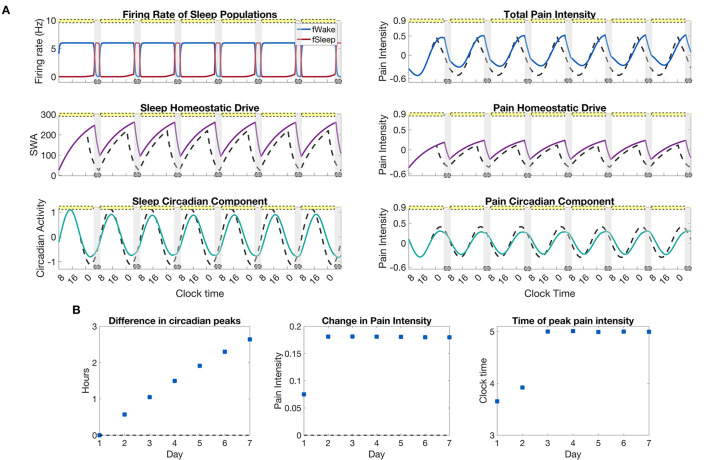
Pain sensitivity due to a restricted sleep schedule of 4 h per night. **(A)**, left panels: output of the SWFF model for restricted sleep from 4–8 a.m. each night for a week. The top panel shows the firing rates of the wake (blue) and sleep (red) populations, the middle panel the sleep homeostatic drive *H*_*s*_, and the bottom panel the circadian rhythm drive to the SCN *C*_*s*_. **(A)**, right panels: output of the pain sensitivity model for the chronic sleep deprivation protocol. The top panel shows total pain intensity, the middle panel the homeostatic component *H*_*p*_, and the bottom panel the circadian component, *C*_*p*_, of pain intensity. Solid curves indicate the sleep restriction protocol while dashed curves represent a simulation with no sleep restriction. **(B)**, left to right: the difference in circadian peaks for each day of the simulation, the average change in pain intensity over the sleep restriction protocol as compared to a normal simulation with no sleep restriction, and the time of the peak in pain intensity for each day. The yellow bars indicate the timing of indoor fluorescent light (600 lx), while the gray bars indicate the timing of sleep as dictated by the SWFF model.

As a final application of the model, we simulate a week of chronic sleep deprivation during which sleep is restricted to the window of 4–8 a.m. each night. Figure 8A shows the model-predicted sleep-wake patterns and pain intensity rhythm, as well as the heightened homeostatic drive and shifted circadian rhythm due to the sleep restriction. As expected, the homeostatic drive rises to elevated levels the day after the first sleep restricted night and remains there for the rest of the week. As a result of the increased exposure to light during extended wakefulness, the circadian rhythm shifts forward in time a little each day while the change in pain intensity reaches a steady state quickly after the first day of sleep restriction; see Figure 8B. While the average amount of pain sensitivity remains relatively constant over the remainder of the week, the model predicts a shift in the timing of peak pain sensitivity from about between 3 and 4 a.m. on the first days to closer to 5 a.m. for the last few days; see Figure 8B, right panel.

## 4. Discussion

We have developed a dynamic mathematical model for predicting pain sensitivity by integrating a physiologically-based model of the sleep-wake regulation network with a data-driven model for pain sensitivity based on experimental human pain sensitivity data (Daguet et al., 2022). The pain sensitivity model incorporates components correlated to both the homeostatic sleep drive and the circadian rhythm. In this way, the model can account for effects on pain sensitivity due to the interactions of sleep homeostatic and circadian modulation, which are well-known to result in non-linear effects on sleep duration (Daan et al., 1984).

We use this model to make predictions on the effect of sleep deprivation and light schedule changes (induced by travel, for example) on pain sensitivity. For sleep deprivation, model results predict that pain sensitivity reaches its maximum the day that sleep deprivation (forced wakefulness) is released, with effects lingering for two more days before reaching baseline values. In exploring different durations of sleep deprivation, model results predicted that total pain sensitivity increased during sleep deprivation and the recovery period with the increase in the amount of hours awake, as expected. However, for some cases pain sensitivity actually decreased for a short period of time following the release of sleep deprivation due to the relative phases of the circadian and homeostatic components of pain.

Next, we simulated shifting the light schedule forward in time (simulating travel eastward) and backward in time (travel westward). We found that although sleep durations shortened for eastward travel compared to westward travel, signifying some sleep deprivation, the total pain sensitivity was lower for eastward travel than for westward travel. This result stems from differences in the relative phases of the circadian and homeostatic components of pain sensitivity. Specifically, during entrainment to the eastward (advanced) light cycle, the decreasing phase of the circadian component can align with the decreasing phase of the homeostatic component resulting in lower average daily pain sensitivity levels. Conversely, for westward travel, the circadian component shifts later in time, with its peak still aligning with the decreasing phase of the homeostatic drive, resulting in larger average pain sensitivity than under normal conditions. Finally, by including sleep deprivation as would occur during flights to the respective locations, we showed that both traveling east and west resulted in an increase in pain sensitivity due to increased homeostatic sleep drive. However, the increase was more striking for westward compared to eastward travel, reflecting a combined effect of changes in light schedules and sleep deprivation.

Finally, we simulated a chronic sleep deprivation scenario in which sleep was restricted to 4 h a night from 4 to 8 a.m. The model predicted increased pain sensitivity for the duration of the sleep restricted week and a forward shift in time of about 1.5 h of the peak in pain sensitivity.

Our model results quantify pain intensity using the same measures as the data reported by Daguet et al. (2022), namely a unitless *z*-score for pain intensity measured by a visual analog scale (VAS). While Daguet et al. suggest that an amplitude change of ~0.6 in these units correlated with a change in pain level of 1/10 on their 100 mm VAS, their results do not provide explicit information to interpret changes in unitless VAS *z*-scores to behaviorally meaningful changes in pain sensation. Additionally, the subjectivity of responses to painful stimuli and in a subject's response in a VAS questionaire increase the difficulty of such an interpretation. However, based on the authors' approximation, model results predict that sleep deprivation and restriction would result in pain sensitivity changes that may be detected on a VAS. Importantly, model results can help understand relative changes in pain sensitivity across different sleep restriction and light schedule changes that can be useful in evaluating potential sleep-wake schedules to accommodate shift work and other non-standard schedules. Further controlled experimental studies quantifying sleep and circadian modulation of pain sensitivity are needed to better interpret the behavioral significance of model predictions.

In this study, we implemented a simple, physiologically-based model for sleep-wake regulation, namely the SWFF that accounts only for the states of wake and sleep (Diniz Behn et al., 2007; Phillips and Robinson, 2007; Postnova et al., 2012; Athanasouli et al., 2022). The SWFF model does not differentiate between rapid eye movement (REM) sleep and non-REM sleep, as in other models of the sleep-wake regulation network (Diniz Behn and Booth, 2010; Gleit et al., 2013; Piltz et al., 2020), nor does it include other brain processes that are known to contribute to sleep-wake transitions such as the orexinergic system, as other models have done (Diniz Behn et al., 2008). However, since the majority of sleep-wake regulation models simulate the homeostatic sleep drive and incorporate circadian modulation similarly to as done here and in other SWFF models, we expect that our framework for modeling pain sensitivity can be adopted to these other sleep-wake models. This would allow analysis of the effects of diverse sleep behaviors and disruptions on pain sensitivity.

While numerous clinical and experimental studies observe a daily cycle of pain sensitivity that is related to circadian rhythms and sleep homeostasis, the direct physiological mechanisms for pain rhythmicity have not been completely identified. There is clear evidence for circadian effects at the level of the spinal cord, particularly within the dorsal root ganglia, which are the neural structures that contain the cell bodies for the sensory afferent neurons transmitting pain signals from the periphery (Kusunore et al., 2010; Zhang et al., 2012). There are equally clear effects for sleep-dependent modulation of the top-down inhibition of pain. Sleep deprivation in humans has been shown to eliminate distraction-based analgesia (Tiede et al., 2010) and decrease central pain modulation (Haack et al., 2009; Campbell et al., 2011). Additionally, pharmacological manipulations that mimic top-down pain inhibition, such as morphine, are ineffective following severe sleep deprivation (Ukponmwan et al., 1984; Nascimento et al., 2007). Finally, the effect of circadian rhythms and homeostatic sleep pressure on pain sensitivity may differ depending on the type of pain measured (Hagenauer et al., 2017).

The model introduced here captures the phenomenon of influences of the circadian rhythm and sleep homeostasis on pain sensitivity without making any assumptions on how these influences originate. Identification of direct physiological mechanisms for the modulation of pain sensitivity by circadian and homeostatic systems will allow for more physiologically-based models of this phenomenon. For example, in previous work we developed a mathematical model of the neural circuitry in the dorsal horn of the spinal cord that processes pain stimuli from the periphery (Crodelle et al., 2019). We accounted for circadian rhythmicity of pain sensitivity through variation of primary afferent responses across the day. Further experimental work identifying the source of circadian and homeostatic influences can inform this and other such models of dorsal horn pain processing circuits (Medlock et al., 2022) in order to understand more fully the complex interactions on pain sensitivity and their potential alleviation with targeted therapeutics.

Our model considers the feedforward modulation of pain sensitivity by the homeostatic sleep drive. However, painful conditions can, in turn, affect sleep behavior. Generally, increased sensitivity to pain during the night is coordinated with the daily sleep-wake cycle to promote rest and healing (Bruguerolle and Labrecque, 2007). But, the presence of pain is arousing and can inhibit sleep, especially the deeper recuperative stages of sleep (Lautenbacher et al., 2006). When sleep is disrupted or limited, the perception of pain may further intensify and pathological processes responsible for the development of chronic pain may be promoted (Finan et al., 2013). This can create a vicious cycle of inadequate sleep and pain management (Lautenbacher et al., 2006). Our modeling formalism is equipped to incorporate bi-directional effects between pain sensitivity and arousal through multiple mechanisms, such as pain-mediated modulation of the sensitivity to the homeostatic sleep drive, for example. Additionally, potential feedback of painful conditions on the circadian rhythm can be accounted for by models of the circadian clock that include effects of non-photic stimuli (St. Hilaire et al., 2007). Further experimental work is needed, however, to collect data on these feedback relationships between pain, sleep homeostasis and the circadian rhythm. Dynamic mathematical models, such as we develop here, can play an important role in analyzing consequences of this vicious cycle, leading to better understanding of the interactions between sleep and pain, and improvements in pain management.

## Data availability statement

Publicly available datasets were analyzed in this study. This data can be found here: https://github.com/jcrodelle/sleepPain.

## Author contributions

JC and VB designed and directed the study. JC and CV performed the numerical simulations. All authors developed the model equations and contributed to writing the manuscript. All authors contributed to the article and approved the submitted version.
